# Identification of rare variants in Alzheimer’s disease

**DOI:** 10.3389/fgene.2014.00369

**Published:** 2014-10-28

**Authors:** Jenny Lord, Alexander J. Lu, Carlos Cruchaga

**Affiliations:** ^1^Department of Psychiatry, Washington University School of MedicineSt. Louis, MO, USA; ^2^Hope Center Program on Protein Aggregation and Neurodegeneration, Washington University School of MedicineSt. Louis, MO, USA

**Keywords:** Alzheimer’s disease, rare variants, exome sequencing, genome sequencing, replication, population differences

## Abstract

Much progress has been made in recent years in identifying genes involved in the risk of developing Alzheimer’s disease (AD), the most common form of dementia. Yet despite the identification of over 20 disease associated loci, mainly through genome wide association studies (GWAS), a large proportion of the genetic component of the disorder remains unexplained. Recent evidence from the AD field, as with other complex diseases, suggests a large proportion of this “missing heritability” may be due to rare variants of moderate to large effect size, but the methodologies to detect such variants are still in their infancy. The latest studies in the field have been focused on the identification of coding variation associated with AD risk, through whole-exome or whole-genome sequencing. Such variants are expected to have larger effect sizes than GWAS loci, and are easier to functionally characterize, and develop cellular and animal models for. This review explores the issues involved in detecting rare variant associations in the context of AD, highlighting some successful approaches utilized to date.

## INTRODUCTION

Alzheimer’s disease (AD), the most common form of dementia, is a devastating and incurable condition affecting over 5.2 million individuals in the US alone (Alzheimer’s Association Statistics, 2014). As populations across the globe age, this figure is expected to increase vastly, posing a huge threat to public health (Brookmeyer et al., 2007). With no cure or effective treatments currently available, the burden of the disease will vastly increase, yet it is thought that just delaying the onset of the condition by a few years could significantly decrease the number of people developing the disorder (Brookmeyer et al., 2007). However, with our current limited knowledge of the etiology of the disease, the appropriate targets and interventions remain unclear. Uncovering genetic loci associated with the condition gives clues to the important pathways and mechanisms involved in the disease process, which can translate to novel targets for diagnoses and treatments.

The rare, familial, early onset form of the condition is comparatively well understood, known to be caused by rare damaging mutations inherited in a Mendelian fashion in three genes (*APP, PSEN1*, and *PSEN2*) related to amyloid processing, which cause a buildup of amyloid in the brain or shift the ratios of the types of Aβ produced. The late onset form, however, is a complex disorder with multiple genetic and environmental risk factors. Although the heritability of the disease is estimated to be around 60–80% (Gatz et al., 2006), our understanding of the genes and loci involved has been limited until relatively recently.

The *APOE* locus was identified as a genetic risk factor for late onset AD (LAOD) in the early 1990s via linkage studies in large AD affected families (Pericak-Vance et al., 1991). As a genetic risk factor for a late onset, complex disorder, *APOE* has an unusually large effect size, which facilitated its discovery by these methods. The risk associated ε4 allele conveys an increase in risk of around 2–3x in heterozygous form, and around 15x in homozygous form, while the ε2 allele is associated with decreased risk (Farrer et al., 1997). Even with such an effect size, the population attributable fraction of *APOE*’s ε4 allele is estimated to be only 27.3% (Lambert et al., 2013), so a large proportion of the heritability remained unexplained. Despite a wealth of research over the following years, further linkage studies as well as numerous candidate gene investigations failed to find any further genetic risk loci for AD that were robust and replicable (Ertekin-Taner, 2010).

## GENOME WIDE ASSOCIATION STUDIES

The next major leap forward came about via the advent of the genome wide association study (GWAS). GWAS enable researchers to test for association between a given disease or trait and virtually all loci within the human genome in a single pass. A caveat of this is that with such a large number of tests being conducted, the sample sizes required to give sufficient power are very large. This technique was facilitated by the coming together of a number of crucial factors. Firstly, international collaborative efforts such as the 1000 genomes project, which set out to catalog all common human variation across multiple populations (Abecasis et al., 2010). This meant in turn that genotyping arrays could be developed that captured the vast majority of human variation in a single array. Finally, and again through large collaborations, sample cohorts of a large enough size to give sufficient power to these studies became available.

The results of the first two truly large scale GWAS in AD were published in October 2009, and brought about the first major advance in the AD genetics field in over a decade. The two studies collectively identified three new loci associated with AD risk, in the genes *CLU*, *PICALM,* and *CR1* (Harold et al., 2009; Lambert et al., 2009). Subsequently, these loci have been extensively replicated and shown to be linked to a variety of AD associated endophenotypes, giving extremely strong evidence for their genuine involvement in the etiology of the condition (Carrasquillo et al., 2010; Corneveaux et al., 2010; Jun et al., 2010; Seshadri et al., 2010). Since then, through ever increasing sample sizes and meta-analyses, a further 17 genes have been reported to be associated with AD via GWAS, most recently in a “mega”-meta-analyses featuring almost 75000 samples (Seshadri et al., 2010; Hollingworth et al., 2011; Naj et al., 2011; Lambert et al., 2013).

While the successes of GWAS in AD are irrefutable, there remain some crucial limitations. Firstly, GWAS can only really detect trait associated common variants – these are the variants included in the widely available genotyping chips used for GWAS to date. Rare variants are poorly targeted by the design. Secondly, although a plethora of loci associated with AD have been identified, the variants underlying these associations remain largely unclear. Often the strongest associated variants within an identified locus fall in the intronic regions of genes, or even in intergenic regions, making it unclear what the actual causative factors in the area are. The early theory that rare, causative mutations were the underlying source of the association being tagged by common GWAS variants have fallen out of favor since in AD as well as numerous other fields of research, extensive resequencing efforts at GWAS loci have proved largely unfruitful (Guerreiro et al., 2010; Hunt et al., 2013). Indeed, where rare, associated variants have been detected within GWAS genes, their effects seem to be independent of the GWAS hit, indicating that different forms of variation within a single locus (e.g., rare coding and common regulatory) can exist and have separate effects on the trait(Bettens et al., 2012).

Aside from not knowing the specific causative factors, the GWAS identified loci individually do not explain a large proportion of disease risk. Each of the loci identified by GWAS affects disease risk only a small amount, so even with *APOE* and all 20 GWAS genes combined, there still remains a large amount of missing heritability (Manolio et al., 2009). Another limitation is the increasingly large sample sizes required to detect the increasingly subtle effects on disease risk that GWAS loci have. With these ever diminishing returns, the remaining unknown heritability is unlikely to be entirely explained by this approach.

## MISSING HERITABILITY

The remaining missing heritability of AD is likely to be complex, and unlikely to be resolvable using a single methodology. While other common variants of low risk undoubtedly remain to be discovered, the likelihood of these explaining the full heritability of AD is low. Non-coding, regulatory variations, rare coding variants of moderate to high effect sizes and epigenetics all likely contribute to AD heritability. Indeed, two articles recently published have presented the findings of the first major epigenome wide association studies in the AD field, and together identified and replicated four loci where epigenetic alterations were associated with AD risk (De Jager et al., 2014; Lunnon et al., 2014), and it is likely more sites where epigenetic modifications relate to AD risk exist (Lord and Cruchaga, 2014).

Although AD is a condition with typical onset far beyond reproductive age, in general, deleterious alleles are more likely to be rare due to the laws of purifying selection (Gibson, 2011), so high impact alleles are unlikely to be detected by GWAS, which target common variants. Furthermore, as rare causative alleles are discovered, they are likely to have a greater explanatory power over the etiology of complex traits than common risk variants do.

Sequencing technologies have seen huge advances in accuracy, throughput, and cost effectiveness in recent years (Cirulli and Goldstein, 2010). Similar to the way in which the development of genotyping arrays facilitated the discovery of common disease associated variants via GWAS, the rapid improvements in sequencing technologies and enrichment strategies over recent years have paved the way for the detection of rare, disease associated variants via exome or whole genome sequencing (WGS).

Despite the huge decrease in sequencing costs, it is still prohibitively expensive for most studies to conduct WGS on large cohorts. WGS is also problematic in terms of data handling, storage, and interpretation. A number of possible strategies are available to circumvent this issue, such as targeted sequencing, exome arrays, exome sequencing, and selection of highly informative subjects, such as members of multiply affected families, specific populations with low heterogeneity, or extremes of phenotypes.

Targeted sequencing, e.g., for loci identified by GWAS, is a cheaper alternative to whole genome or whole exome sequencing (WES), and has had some successes (Rivas et al., 2011). Although it has been attempted for several of the early AD GWAS hits, it proved largely unfruitful, as has been seen to be the case in other complex disorders (Guerreiro et al., 2010; Ferrari et al., 2012; Hunt et al., 2013).

Exome arrays offer a platform for genotyping exonic low frequency and rare variants in a chip based assay, bridging the gap between the sparse genotyping of GWAS and sequencing based study designs. A number of commercially available exome arrays are available, often allowing for the incorporation of custom content to tailor the platform to specific diseases or traits. The heterozygous concordance rate between exome sequencing data and exome array data has been estimated to be 98.14% (Wang et al., 2013). Exome array based studies have been shown to be successful in identifying several loci associated with insulin levels in a cohort of 8229 Finnish non-diabetic individuals (Huyghe et al., 2013). However, there are issues with the technology, and to date, no variants associated with AD have been published using the exome array method. Chung et al. (2014) used the Affymetrix Axiom Exome Genotyping Array in their study of 1005 Korean subjects, but the only significant association signals were found to be due to *APOE*. One of the reasons for this may be that the selection of variants for exome arrays was mainly based on individuals of European origin, so their utility in other populations may not be great. The sample size utilized here was also significantly smaller than Huyghe et al.’s (2013) design, making it unclear whether the lack of significant associations were due to genetic differences in the sample population compared to the chip design, or a simple lack of power. The inclusion of variants in exome arrays is also dependent on their genomic context, not all are compatible with the array type genotyping design (Do et al., 2012). Exome arrays also do not offer the ability to detect previously unknown variants, which targeted or exome sequencing designs do. Exome sequencing, although only targeting 1–2% of the genome, targets the most commonly disease associated regions (Ng et al., 2009). Additionally, it lessens the issues associated with data handling, processing and storage, as well as being easier to interpret (as bioinformatics programs for prediction of consequences of exonic mutations are generally more advanced than those for non-coding changes), and is also cheaper, enabling larger sample sizes to be utilized than would be possible with WGS.

The power of association tests between single variants and traits decreases as the minor allele frequency (MAF) of the variant does. This means very large samples will be needed for rare variant association studies unless the effect size of the variant is particularly large. It is also not clear at present what the most appropriate way to correct for multiple tests in rare variant studies is. The typical GWAS significance threshold of 5×10^-8^ is based on approximately one million independent tests being conducted simultaneously. Although rare variants are unlikely to be completely independent, there are far more rare variants than GWAS genotyped common variants, so the required significance threshold could be even more stringent, making attaining significance in single variant analyses problematic. Evaluating multiple rare variants within a given region (e.g., a gene) can help combat this. A number of methods have emerged over the last few years to enable this. The simplest of these are burden tests, but these do not account for variants in the same test unit having opposing directions of effects, which can cancel out any meaningful signals (Morris and Zeggini, 2010; Lee et al., 2014). Indeed, recent evidence from the Alzheimer’s field suggests many loci do have variants which both increase and decrease disease risk (e.g., the protective alleles recently reported in *APP* and *APOE*, both long standing AD risk loci harboring deleterious variants’ Jonsson et al., 2012; Medway et al., 2014). Combined tests, such as SKAT-O can take in to account both risky and protective variants within the same locus, aggregating the individual association signals in to a single combined association score, which can be much more powerful than the single variant approach (Lee et al., 2012, 2014).

Selecting extremes of the phenotypic spectrum has promise for rare variant discovery since such individuals are likely to be enriched for rare disease causing variants. In terms of AD, this could be particularly severe cases or those with early onset vs. cognitively healthy extremely old individuals. It is also possible to use levels of biomarkers, which typically follow normal distributions, and conduct sequencing in the highest and lowest measuring individuals. Using quantitative traits as endophenotypes has been shown to have increased power compared to standard designs, reducing the number of individuals to be sequenced substantially (Li et al., 2011; Benitez et al., 2013b). Another potential way to enrich subjects for genetic risk factors is to utilize individuals with a strong family history of the condition, since pedigrees with multiple affected individuals are more likely to have pre-disposing genetic variants than typical sporadic cases. Indeed, the way in which traditional genetic linkage approaches can be effectively used in conjunction with new technological and analytical methods for the identification of rare, disease causing mutations is currently being explored (Santorico and Edwards, 2014).

It is likely that, as with GWAS, combining several individually smaller studies in to one large meta-analysis will prove an effective way of increasing sample sizes, and may produce findings beyond those of the separate studies alone. At present, however, there is little standardization in the analysis of next generation sequencing data across studies, which could make combining them problematic. Methods will need to be developed to adequately deal with heterogeneity between studies, particularly given the high variance in rare variant detection and quantification across different sequencing platforms and analysis strategies (O’Rawe et al., 2013).

Another strategy for increasing power without affecting costs is to utilize imputation, inferring genotypes in unsequenced individuals to increase effective sample size. However, the accuracy of imputation decreases as MAF decreases, so imputation of rare variants is unreliable at present for unrelated individuals. As more sequencing data, both whole genome and exome, is generated, the reference panels upon which imputations are based will become more extensive, making an improvement in imputation accuracy for rare variants likely in the coming years. Additionally, several methods are being developed to infer the genotypes of rare variants in related individuals, using sequencing data, GWAS data, and pedigree information (Cobat et al., 2014; Saad and Wijsman, 2014).

A further complication is that rare variant studies are more susceptible to cross population differences in allele frequency. Rare variants, which are more likely to be recent in origin, can show vast differences in frequencies across populations (Raska and Zhu, 2011). Whilst this can be leveraged to be advantageous (Hatzikotoulas et al., 2014), it also provides difficulties, since subtle underlying population substructure can inflate false positive rates (Keen-Kim et al., 2006). Although robust methods exist for correcting for such stratification in GWAS data, the same cannot currently be said for rare variant association studies (Liu et al., 2013). These matters will be discussed in the context of rare variant association studies in AD below.

## RARE VARIANTS AFFECTING AD RISK

Despite being faced with this plethora of issues, in recent years several research groups have had success in identifying low frequency and rare variants associated with AD, with some variants and genes yielding protective effects, and others increasing AD risk.

### TREM2

One of the first reports of AD associated rare variants utilizing the recent advances in sequencing technologies was Jonsson et al.’s (2013) study, which found that the rare missense variant in TREM2, rs75932628 [predicted to encode the protein change, R47H, with NHLBI Exome Sequencing Project (ESP, http://evs.gs.washington.edu/EVS/) MAF of 0.26% in European–Americans (EA) and 0.02% in African–Americans (AA)] conveyed an increased risk of AD in the Icelandic population (Jonsson et al., 2013). From 2261 Icelandic individual’s whole genome sequence, the group identified 191777 variants likely to be affecting protein function across the genome. These variants were imputed in to an AD case control cohort (3550 AD, 8888 controls), and rs75932628 was the only variant to exceed the applied Bonferroni correction for multiple tests, other than those at the *APOE* locus (*p* = 3.42 × 10^-10^, odds ratio (OR) = 2.92 [95% confidence interval (CI) 2.09-4.09)]. The use of imputation at this stage allowed the 191777 potentially functional variants to be narrowed down to just one likely AD associated variant. In a further four replication cohorts of European origin, the variant was seen to convey an increased risk of AD, with a combined sample size of 2037 AD and 9727 controls giving compelling evidence for R47H’s involvement in AD risk [*p* = 0.002, OR = 2.83 (95% CI 2.16 - 3.91)].

Simultaneously with Jonsson et al.’s (2013) TREM2 rare variant report, a second report of R47H’s involvement in AD risk was published by Guerreiro et al. (2013). This group approached the gene as a biological and statistical candidate, citing its relationship with the recessive early onset dementia and bone cyst disease, Nasu-Hakola; the identification of homozygous TREM2 mutations in three Turkish patients with a frontotemporal dementia like syndrome; and evidence of a nominally significant linkage association between a region on chromosome 6 containing the gene and risk of AD. Using whole exome, whole genome and Sanger sequencing data from 1092 AD patients and 1107 control samples, the group was able to demonstrate the presence of an excess of variants in the second exon of TREM2 in AD relative to controls (*p* = 0.02), as well as a number of variants found exclusively in either case or control samples. The variant encoding R47H (rs75932628) showed significant association with AD (*p*< 0.001). These findings were replicated by imputing the variant in three GWAS datasets (totaling ∼5500 AD cases and >13,000 controls, *p* = 0.002). In an additional replication stage conducted by directly genotyping rs75932628 in 1887 AD patients and 4061 controls, the variant again showed a strong, significant association with AD [OR 4.59 (95% CI 2.49 - 8.46), *p* = 1.4 × 10^-7^].

Jin et al. (2014) conducted a resequencing project of TREM2 in 2082 AD patients and 1648 controls of EA origin. A total of 16 non-synonymous variants were detected in the gene, six of which had not been reported in connection with AD previously. As well as replicating the association seen between R47H and AD (see **Table 1**), an additional variant, predicted to encode the protein change R62H was also associated with AD [OR = 2.36 (95% CI 1.47 - 3.80), *p* = 2.36 × 10^-4^]. Additional replication for the association between R47H and AD risk has been provided in subjects of French origin (see **Table 1**; Pottier et al., 2013), as well as suggestive association with R47H and R62H in the Belgian population, where a gene based association test attained statistical significance [relative risk = 3.01 (95% CI 1.29 - 11.44), *p* = 0.009; Cuyvers et al., 2014], strengthening the evidence for TREM2 ’s association with AD in subjects of European origin.

**Table 1:** Association between TREM2 variant R47H and AD risk.

Study	Population	Sample size	OR (95% CI)	*P* value
Jonsson (Jonsson et al., 2013)	Icelandic	3550 AD, 8888 controls	2.92 (2.09-4.09)	3.42 × 10^-10^
Guerreiro (Guerreiro et al., 2013)	European and European American (EA)	1091 AD, 1105 controls	4.5 (1.7-11.9)	<0.001
		1994 AD, 4062 controls	5.05 (2.77-9.16)	9.0 × 10^-9^
Jin (Jin et al., 2014)	EA	2082 AD, 1648 controls	2.63 (1.44-4.81)	9.17 × 10^-4^
Pottier (Pottier et al., 2013)	French	726 EOAD*, 783 controls	4.07 (1.3-16.9)	0.009
Cuyvers (Cuyvers et al., 2014)	Belgian	1216 AD, 1094 controls	3.01 (0.83-10.94)	0.08
Benitez (Benitez et al., 2013a)	Spanish	180 EOAD*, 324 AD, 550 controls	Seven heterozygous cases found, no heterozygous controls	0.009
Yu (Yu et al., 2014)	Han Chinese	1133 AD, 1159 controls	R47H not present	-
Jiao (Jiao et al., 2014)	Han Chinese	360 AD, 400 controls	R47H not present	-
Miyashita (Miyashita et al., 2014)	Japanese	2190 AD, 2498 controls	Not associated, three heterozygotes found (one case, two controls)	-

Summary of the study designs and findings from reports of R47H TREM2 variant and AD risk.

*EOAD, early onset Alzheimer’s disease.

In subjects of Asian origin, the same replication has not been seen. Two large studies in the Han Chinese population (totaling > 3000 samples) did not find the R47H variant (Jiao et al., 2014; Yu et al., 2014), and no association was seen between the variant and AD in a Japanese cohort of 2190 AD cases and 2498 controls (see **Table 1**). It is not clear whether there are other variants within TREM2 in these populations that affect AD susceptibility.

These studies demonstrate that while particular risk variants may be population specific, the same genes can harbor different disease associated variants. Rather than genotyping associated variants in follow up studies in different populations, a more appropriate approach may be to resequence the genes, enabling the detection of population specific variants which would otherwise be overlooked. Another notable fact is that even within a traditionally accepted “single population,” such as Europeans, there is substantial regional variance in allele frequency among rare variants, which is evidenced by the widely varying ORs observed for different cohorts of European origin in **Table 1**.

### PLD3

Cruchaga et al. (2014) utilized a family based study design which enabled the detection of rare variants within the PLD3 gene associated with AD risk. The group conducted WES on individuals from large LOAD affected families, and looked for rare variants perfectly segregating with disease status in the sequenced members as well as additional genotyped family members, and sought association in a large independent case-control cohort. The selection of large families with multiple affected individuals, prioritization of earlier ages of onset, and exclusion of families where *APOE* allele ε4 perfectly segregated with disease was designed to give a cohort of related samples enriched for genetic risk factors for LOAD. The variant rs145999145 (predicted to encode a Val232Met alteration in protein sequence, ESP MAF 0.49% in EA, 0.25% in AA) segregated with disease status in two independent families. When the variant was genotyped in 4998 cases and 6356 EA controls, the variant showed strong association with disease status [OR 2.10 (95% CI 1.47-2.99), *p* = 2.93 × 10^-5^], replicating the initial finding. In search of further risk associated variants within the PLD3 gene, the group sequenced the gene’s coding region in 2363 cases and 2024 controls of European descent, as well as 130 cases and 172 controls of AA descent. In the European subjects, 14 variants more common in cases than controls were detected, including nine exclusively found in cases. Using SKAT-O to conduct a gene based burden test allowed the group to demonstrate again a significant association with disease risk [OR 2.75 (95% CI 2.05 - 3.68), *p* = 1.44 × 10^-11^], which remained significant when the initial variant, rs145999145, was excluded from the analysis [OR 2.58 (95% CI 1.87 - 3.57), *p* = 1.58 × 10^-8^], indicative that the locus harbors additional variants impacting on disease risk within this population.

In the AA samples, rs145999145 as well as another variant nominally associated in European subjects (predicted to be a synonymous variant, Ala442Ala, affecting splicing and gene expression) were observed in cases but not controls, with Ala442Ala showing significant association with AD in this cohort (*p* = 0.03). Gene based analyses in this cohort also revealed a significant association between PLD3 and AD risk [OR 5.48 (95% CI 1.77-16.92), *p* = 1.4 × 10^-3^]. Again, this highlights the heterogeneity of genetic risk factors for AD between populations, and shows that different variants within the same locus may have differing effects (or differing statistical power) in different populations.

### *APP* (PROTECTIVE)

With *APP* as a clear biological candidate for involvement in AD risk, Jonsson et al. (2012) sought low frequency and rare mutations in whole genome sequence from 1795 Icelanders, and imputed recurrent variants in 71,743 chip-genotyped Icelanders and 296,496 relatives of genotyped individuals. The variant showing strongest significance was rs63750847, predicted to cause an amino acid substitution (A637T, ESP MAF 0.01% in EA, not recorded in AA) at the second position in the Aβ peptide region of *APP*. The variant was reported to be significantly more common in elderly healthy controls than AD subjects (OR 5.29, *p* = 4.78 × 10^-7^), indicative of a protective effect against the development of AD. Furthermore, the protective A allele of the variant was also found to be associated with increased performance on cognitive tests in elderly cognitively normal participants (*p* = 0.0021), suggesting the protective effect of the variant is not limited to AD pathogenesis, but affects cognition in individuals within the healthy spectrum as well.

Although there is some evidence A637T may have a protective role in the Finnish population (Kero et al., 2013), extensive resequencing efforts in white subjects from the U.S. (>4300 individuals) as well as Asian subjects (>11,000 individuals) have found the variant to be absent in those populations (Ting et al., 2013; Bamne et al., 2014; Liu et al., 2014).

### ADAM10

Again, pursued as a potential biological candidate due to its activity as an α-secretase capable of blocking the amyloidogenic processing of *APP*, Kim et al. (2009) sought association between variants in ADAM10 and AD. Nine common “tag” SNPs within the gene were genotyped in over 400 families (995 cases and 411 controls) from The National Institute of Mental Health (NIMH) cohort, with one of the variants (rs2305421) showing evidence of association with AD (*p* = 0.003). When the data was stratified by *APOE* genotype, this variant’s association with the disease was strengthened, and two further ADAM10 variants (rs605928 and rs4775083) showed suggestive association with AD (*p* = 0.02 and 0.06, respectively). None of these variants are observed in NHLBI’s ESP. Although no significant associations between the SNPs and AD were observed in the Consortium of Alzheimer’s Genetics (CAG) cohort, the smaller size of this sample (222 cases and 267 controls) renders it possible the lack of association was an issue of power. The coding regions and flanking non-coding regions of ADAM10 were then Sanger sequenced in individuals from 32 NIMH families where rs2305421 genotype was related to AD status. Two rare, non-synonymous variants were detected in exon 5 of ADAM10, which was then sequenced in the remaining NIMH families, giving a total of three families with the predicted Q170H mutation, and two with R181G. The combined association of these variants with AD was statistically significant (*p* = 0.0043).

However, subsequent research investigating the role of ADAM10 variants in AD did not find evidence for the gene’s involvement (Cai et al., 2012). Additional research will be needed to resolve the gene’s relationship with AD.

### *AKAP*9

Logue et al. (2014) conducted exome sequencing in seven individuals from AA families with multiple AD affected individuals. With 88,867 variants identified, the group adopted a filtering strategy, prioritizing the follow up of variants based on several factors, including novelty (absence from dbSNP 132), the predicted nature of the variant (with non-synonymous changes prioritized), and those in genes or pathways previously related to AD pathogenesis. Of the 44 SNPs genotyped in the first follow up cohort (including ∼400 AA cases and controls), two rare SNPs within the gene *AKAP9* showed nominally significant associations with AD in single SNP association tests (rs144662445, with *p* = 0.014 and OR 8.4 and rs149979685, *p* = 0.037. ESP MAFs for these variants are 0.43 and 0.36% in AA, respectively, and are not observed in EA samples). These were then genotyped in a second AA cohort (1037 cases, 1869 controls) where each of the associations was replicated (*p* = 0.0022, OR = 2.75 for rs144662445 and *p* = 0.0022 with OR = 3.61 for rs149979685). Bioinformatic analyses of the two variants (using SIFT, PolyPhen2, and MutPred) suggested rs144662445 was likely to be benign, while rs149979685 may be a protein function altering causative mutation. The two variants were not present in >4000 Caucasian individuals (ESP and 1000 genomes project) or >280 individuals of East-Asian origin (1000 genomes project), so whether the *AKAP9* gene will harbor rare causative variants in other populations remains to be established.

### UNC5C

The approach adopted by Hunkapiller et al. (2013) used linkage analysis in a large LOAD pedigree showing apparent autosomal dominant inheritance to prioritize areas of the genome likely to be harboring explanatory variants. The group conducted whole genome and WES, each in one sample, selecting the most distantly related individuals to minimize the shared genetic component, and thus the number of candidate causative variants. Detected variants were excluded if they were not within the five identified linkage areas, were non-exonic, homozygous, or fell in areas of segmental duplication, leaving just two candidate missense variants which were shared between the two sequenced members, in the genes *AKAP9* and UNC5C. Although, as discussed above, *AKAP9* has been identified as an AD risk gene in the AA population, when the *AKAP9* SNP rs1063242 was genotyped in 4533 cases and 20,325 controls of European origin, no evidence of association was seen (*p* = 0.54). For the rare >UNC5C variant, however (rs137875858, T835M, ESP MAF 0.06% in EA, 0.02% in AA), a second pedigree was identified in which the variant segregated with disease status, leading this variant to be genotyped in a series of independent cohorts, totaling 8050 cases and 98194 controls. In a combined analysis the variant showed significant association with disease status [OR = 2.15 (95% CI 1.21 - 3.84),*p* = 0.0095].

### *APOE* (PROTECTIVE)

Medway et al. (2014) used an innovative approach to investigate rare variants within the *APOE* locus. By identifying rare variants present in the EVS database, they were able to circumvent the need for costly resequencing. The group identified three variants (L28P/rs769452, R145C/rs769455, and V236E/rs199768005) with MAFs of 0.17, 0.026, and 0.12%, respectively. These were then genotyped in up to 9114 individuals, allowing their relationship with AD to be investigated. R145C proved too rare to be adequately tested for association with AD in the cohort, while L28P was revealed to be in complete LD with the ε4 allele and conveyed no increase in risk beyond that of the ε4 allele itself. The third variant, V236E, although in complete LD with the ε3 allele of *APOE*, gave a decrease in AD risk independent of *APOE* genotype. Indeed, the group highlighted a rare haplotype termed ε3b, which harbored this variant and was significantly associated with a decrease in AD risk comparable to that of the ε2 allele [ε3b OR = 0.1 (95% CI 0.02 - 0.35), *p* = 2.16 × 10^-3^].

The approach utilized here, mining publically available sequencing data for rare variants in disease associated loci, followed by genotyping in a large case-control cohort proved a powerful and cost-effective method to detect this rare, protective variant in *APOE*. A similar approach could be applied to other AD associated loci, including those identified by GWAS, other rare variant studies, or potential biological candidates. A limitation of this approach is that it does rely on previously identified and cataloged rare variants, so no new variants will be identified by this approach. Furthermore, although the *APOE* locus harbored a small amount of rare variants, which made this study feasible, this will not be the case for all loci, and a much greater number of variants may be found in more variable regions of the genome.

### SORL1

SORL1 (also known as *LR11*) was first implicated in AD in 2004, when Scherzer et al. (2004) demonstrated a reduction in its expression in AD relative to controls. Pottier et al. (2012) conducted exome sequencing in 14 subjects with autosomal dominant early onset AD and no mutations in *APP*, *PSEN1,* or *PSEN2*. Five individuals were found to have mutations in SORL1 (one nonsense, four missense), which were not present in 1500 control subjects. In a replication cohort, featuring a further 15 individuals, an additional missense and nonsense mutation were detected, taking the total SORL1 mutation carrying individuals to 7/29. Whilst it is likely that the utilization of an extreme phenotype form of AD facilitated the discovery of such a high frequency of likely deleterious mutations within SORL1 in this study, SORL1 was also one of the genes identified as a risk factor for LOAD in the recent GWAS meta-analysis (Lambert et al., 2013). This suggests the same loci may contribute to both early and late onset forms of the condition, and studies utilizing more extreme forms of the disease, which have increased power, may be informative about risk factors for the late onset, complex form of AD.

A summary of the major findings in each of these studies is presented in **Table 2**.

**Table 2:** Summary of main findings from rare variant reports in AD.

Study	Design	Population	Sample size	Variant/gene	MAF % (EA/AA)	OR (95% CI)	*P* value
Cruchaga – **PLD3** (Cruchaga et al., 2014)	Family based WES, genotyping, and targeted sequencing.	EA (genotyping)	4998 cases, 6356 controls	V232M/rs145999145	0.4884/0.2497	2.1 (1.47–2.99)	2.93 × 10^-5^
		European (sequencing)	2363 cases, 2024 controls	Gene based	N/A	2.75 (2.05–3.68)	1.44 × 10^-11^
		AA (sequencing)	130 cases, 172 controls	Gene based	N/A	5.48 (1.77–16.92)	1.4 × 10^-3^

Jonsson – ***APP*** (Jonsson et al., 2012)	WGS, variants imputed in large cohort.	Icelandic	71,743 chip genotyped individuals and 296,496 relatives	A637T/rs63750847	0.0116/ 0.0	5.29	4.78 × 10^-7^

Kim – **ADAM10** (Kim et al., 2009)	Genotyping and targeted sequencing.	EA	400 families (995 cases, 411 controls)	rs2305421	Not in EVS	–	0.003
				Gene based	N/A		0.0043

Logue – ***AKAP9*** (Logue et al., 2014)	Family based WGS, Genotyping.	AA	1037 cases, 1869 controls	rs144662445	0.0/0.4312	2.75	0.0022
				rs149979685	0.0/0.3631	3.61	0.0022

Hunkapiller – **UNC5C** (Hunkapiller et al., 2013)	Family based WGS and WES, Genotyping.	EA	8050 cases, 98194 controls	T835M/rs137875858	0.0581/0.0227	2.15 (1.21–3.84)	0.0095

Medway – ***APOE*** (Medway et al., 2014)	EVS database, Genotyping.	EA/ European	4128 cases, 4986 controls	V236E/rs199768005	0.1188/0.0	0.1 (0.03–0.45)	7.5 × 10^-5^

Pottier – **SORL1** (Pottier et al., 2012)	WES in EOAD	European origin	29 cases, 1500 controls	7/29 AD carried variants, no controls did		

*Summary of the study designs and major findings from the discussed rare variant association studies in the AD field. WGS, whole genome sequencing; WES, whole exome sequencing; EOAD, early onset Alzheimer’s disease; EA, European American; AA, African American; MAF, minor allele frequency. MAFs taken from NHLBI’s Exome Sequencing Project (EVS, Exome Variant Server; http://evs.gs.washington.edu/EVS/, accessed September 2014).*

## FINAL COMMENTS

One of the major lessons that can be learned from these rare variant association studies in AD so far is that population stratification can have a profound affect both on findings in initial reports and replication. Large sample sizes are required for replication in order to distinguish whether a failure to replicate is due to a lack of power or a lack of association. A lack of association may indicate the initial finding to be a false positive result, or highlight differences in the etiology of the disease between the discovery population and the replication cohort. Replication in both the same and alternative populations is crucial, particularly at the gene rather than variant level, since associated variants can differ between populations.

The overriding aim of all of these studies is to better understand the etiology of AD. Identification of new genes and variants associated with AD will bring new targets for disease diagnostics and treatments. A number of major resequencing efforts are being undertaken in AD, with significant insights into disease biology likely from their results.

## Conflict of Interest Statement

The authors declare that the research was conducted in the absence of any commercial or financial relationships that could be construed as a potential conflict of interest.
